# Bumblebee flower constancy and pollen diversity over time

**DOI:** 10.1093/beheco/arad028

**Published:** 2023-04-10

**Authors:** Johanna Yourstone, Vidula Varadarajan, Ola Olsson

**Affiliations:** Department of Biology, Lund University, Sölvegatan 37, 223 62 Lund, Sweden and; School of Arts and Science, Azim Premji University, Survey No 66, Burugunte Village, Bikkanahalli Main Road, Sarjapura, Bengaluru 562125, India; Department of Biology, Lund University, Sölvegatan 37, 223 62 Lund, Sweden and

**Keywords:** *Bombus*, foraging, flower fidelity, foraging preferences, seasonal shift

## Abstract

Bees often focus their foraging effort on a few or even a single flower species, even if other equally rewarding flower species are present. Although this phenomenon—called flower constancy—has been widely documented during single foraging trips, it is largely unknown if the behavior persists over longer time periods, especially under field conditions with large temporal variations of resources. We studied the pollen diet of individuals from nine different *Bombus terrestris* colonies for up to 6 weeks, to investigate flower constancy and pollen diversity of individuals and colonies, and how these change over time. We expected high degrees of flower constancy and foraging consistency over time, based on foraging theory and previous studies. Instead, we found that only 23% of the pollen foraging trips were flower constant. The fraction of constant pollen samples did not change over the study period, although repeatedly sampled individuals that were flower constant once often showed different preferences at other sampling occasions. The similarity of pollen composition in samples collected by the same individuals at different occasions dropped with time. This suggests that the flower preferences change in response to shifting floral resources. The average diversity of pollen from single foraging trips was around 2.5 pollen types, while the colony-level pollen diversity was about three times higher. How rapidly preferences change in response to shifting resources, and if this differs between and within bee species depending on factors such as size, should be the focus of future research.

## Teaser

Foraging bumblebees often focus on certain flowers. By studying pollen foraging, we show that this focus is not long-lasting in the field, and that the visited flower species vary over time. Only in less than a quarter of the cases, buff-tailed bumblebee individuals focused on certain flowers during foraging trips. Instead, most individuals collected several types of pollen. The more pollen types each individual collected, the more pollen types were collected to the bumblebee colony.

## INTRODUCTION

For most animals foraging consists of a sequence of decisions, for example, in terms of what areas to visit, what resources to use, how long to stay, and when to rest. These decisions determine resource intake rates, energy expenditure, and predation risk, and so, ultimately fitness (e.g., Lemon 1991; Olsson et al. 1999; van Gils et al. 2009). Thus, foraging strategies should be optimized by natural selection (Stephens et al. 2007), but may also be constrained by physical and perceptual limitations (e.g., Raine and Chittka 2007b; Olsson and Brown 2010). As food is often heterogeneously distributed and varies in abundance, foraging strategies are context dependent. For example, animals that forage on pollen and nectar from flowers have to cope with a spatial, and often a temporal and morphological variation of resources, which requires motor learning (for resource acquisition from specific flowers), memorizing of rewarding resources, and navigational skills (Menzel and Erber 1978; Chittka 1998; Raine and Chittka 2007b; Collett et al. 2013). One strategy that reduces the complexity of foraging is to specialize in one or a few specific food items, which is commonly seen across animal taxa (Heinrich 1976; bumblebees, Rausher 1978; butterfly, Pietrewicz and Kamil 1979; bird). This behavior has long been studied in bumblebees foraging for nectar and pollen in flowers and has intrigued researchers since Darwin’s time (e.g., Darwin 1876; Grant 1950; Free 1963, 1970; Heinrich 1976). The behavior has been termed *flower constancy* and is defined as a preference for one or a few flower resources, even though other equally rewarding resources are available Waser (1986).

Flower constancy has been explained by constraints or costs related to the acquisition of new or multiple resources, such as limitations in the short-term memory (Raine and Chittka 2007a) including memory interference between recently and previously learned species (Darwin 1876; Chittka and Thomson 1997; Gegear and Laverty 1998), costs of learning to recognize and handle new resources (Dukas and Real 1993), and costs of sampling alternative resources (Chittka et al. 1999; Grüter and Ratnieks 2011). There is no current consensus on the reasons for constancy (Grüter and Ratnieks 2011), however, models and experimental data suggest that the behavior is energetically beneficial for bumblebees, especially when resources are abundant (Gegear and Thomson 2004; Hayes and Grüter 2022).

It is not well-known how long flower constancy lasts in nature, where floral resources are often diverse, change over time, and are distributed over very different spatial scales than in a lab. Most studies of constancy behavior have been done in laboratory settings, and most of them over only short time periods (e.g., Waser 1986; Dukas and Real 1993; Chittka et al. 1997; Austin et al. 2019, but see Martínez-Bauer et al. 2021). It has been shown that bees can have delayed responses to declining resources in foraging patches they return to (Thomson 1981b), and life-long flower constancy in a few, but not all, bumblebee individuals was observed in laboratory settings (Russell et al. 2017). These findings indicate behaviors that are not (at least instantly) adapting to environmental conditions. Long-term flower preferences might be disadvantageous if flower visitors stick to a declining resource when other flowering species present would provide more rewarding foraging, and thus deserve attention.

Bumblebees are a group of large-bodied, central-place foraging, and social bees, that often display flower constancy on an individual level (Heinrich 1976; Thomson 1981a), while they collectively may gather a high diversity of pollen in their colonies (Leonhardt and Blüthgen 2012). They are a good model system to study flower constancy and other foraging behaviors as they feed exclusively on flowers, consuming energy-rich nectar and protein-rich pollen (Vaudo et al. 2015), are relatively easy to follow and sample because of their size, and some species are reared commercially. Pollen foraging is particularly interesting to study as it takes longer to learn compared to nectar foraging (Raine and Chittka 2007b) and there are indications that pollen foraging implies an even more constant behavior than nectar foraging (Rossi et al. 2015).

Collection of diverse pollen is known to support greater bumblebee colony fitness (Brodschneider and Crailsheim 2010; Hass et al. 2018; Schweiger et al. 2022), likely by increasing the chances for an appropriate nutritional balance (Moerman et al. 2017; Watrobska et al. 2020). Thus, colonies are facing the paradox that while individual bumblebees may be flower constant, a high pollen diversity should be aimed for at the colony level. Flower constant individuals within a colony, therefore, need to specialize in different resources to sustain the diversity collected by the colony. It may also be in the interest of individuals to specialize, as a way of reducing competition with other workers foraging in the same landscape, especially if there are costs associated with using many or new flower types (Chittka and Thomson 1997). Indications of resource partitioning among workers from the same colonies have, indeed, been observed in bumblebees (Johnson 1986; Pasquaretta et al. 2019). However, it is not fully established whether the pollen diversity collected by colonies is sustained through individuals specializing in different resources or by individuals collecting a diversity of pollen themselves. For example, Gervais et al. (2020) and Martínez-Bauer et al. (2021) show high individual pollen diversities collected by bumblebee workers over single foraging trips, which is in contrast to rather high degrees of individual constancy per foraging trip seen in other studies (e.g., Leonhardt and Blüthgen 2012; Rossi et al. 2015; Russell et al. 2017).

In this study, we explore the pollen diet of colonies and individuals of *Bombus terrestris* under natural field conditions in agricultural landscapes, with a focus on flower constancy and pollen diversity, to increase the understanding of their foraging behaviors and preferences over time in natural settings. Specifically, we aim to answer the following questions:

1) How flower constant are bumblebee individuals during single pollen foraging trips, and does it change with time?
2) Is the pollen composition collected by individuals sustained over time?
3) How is the pollen diversity collected by individuals affected by time, and how is it related to that of colonies?

Based on previous field studies, we hypothesize that individuals will show a high degree of flower constancy (Leonhardt and Blüthgen 2012; Somme et al. 2015), even if probably lower than in lab conditions (e.g., Gegear and Thomson 2004; Russell et al. 2017). Second, we hypothesize that individuals stick to resources over time (Thomson 1981b; Russell et al. 2017), such that pollen samples from the same individuals on average are more similar than samples from different individuals. Although, because of changing resources and expected exploration behaviors (Lihoreau et al. 2010; Kembro et al. 2019), we also hypothesize that pollen sample similarity goes down with time both within and between individuals. Regarding our third question, we have adopted an explorative approach, as existing theory and data do not guide clear a priori hypotheses. If individuals are specialized in complementary ways to promote an optimal colony pollen diversity, we may expect no relationship between individual and colony diversity. However, even with complementary specialization, the environmental variation in the distribution and diversity of resources may lead to a relation between individual and colony diversity. By exploring pollen diversity on individual and colony levels, as well as over time, we hope to provide useful information for future research on bumblebee foraging ecology.

## METHODS

### Study setup and study area

**Figure 1:**
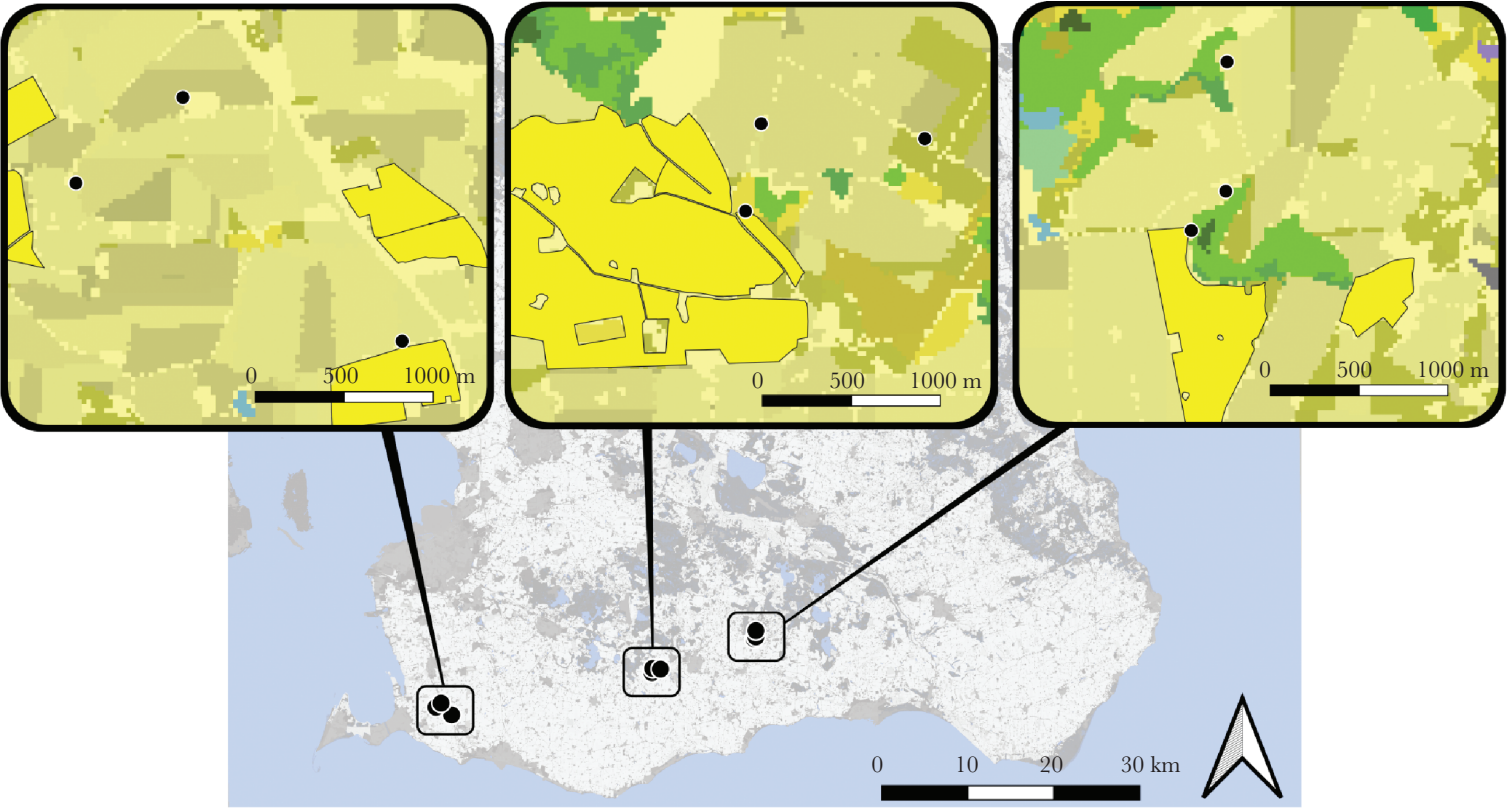
Locations of bumblebee colonies (black dots) placed in farmland in southernmost Sweden, in relation to the nearest oilseed rape fields (bright yellow fields). Bright green fields indicate woody areas and olive-toned fields indicate farmland. Made with QGIS.

Field data were collected in May and June 2017. In the first week of May nine commercial *Bombus terrestris* colonies (BioProduction®, Tappernøje, Denmark) were distributed in three areas in southern Sweden (Figure 1). The bumblebee colonies, each contained in a plastic box with a mesh roof inside a cardboard box, were placed inside protective, ventilated wooden boxes. We took advantage of the study design in Yourstone et al. (2021), using a subset of their sites for bumblebee monitoring. The study design included an oilseed rape (*Brassica napus*) contrast, in which bumblebee colonies were placed at three different distances from the nearest oilseed rape field: adjacent to, 300 m from, or 1000 m from the nearest oilseed rape field—with each of the distances represented once in each study area. We include distance to oilseed rape in the analyses (see below) but refrain from drawing any strong conclusions regarding its impact, as replication was low. The oilseed rape started to flower roughly the 10th of May and flowered for about 1 month. Additionally, as part of Yourstone’s et al. (2021) study design, there was a second bumblebee colony placed immediately next to each of our study colonies (with the entrance facing the opposite direction), but as we saw very few signs of interference among the bees from the two neighboring colonies and as this was affecting all our study colonies the same way, we ignore it.

The study areas were characterized by intensive agricultural land use, where the amount of cropland and semi-natural habitat within 500 m from the bumblebees (summarized per study area) ranged between 69–87% and 4–18%, respectively.

### Field data collection

Monitoring consisted of marking individual workers and sampling their pollen loads (if present). At the first sampling occasion, bumblebees returning to the nests were caught and marked by gluing a number plate (honeybee queen marker) onto their thorax using cyanoacrylate glue, and the pollen from their corbicula was collected if present. The bumblebees were captured by blocking the entrance of their nest and capturing any incoming individual in an entrance tube we had mounted on the nests. At subsequent sampling occasions both already marked bumblebees and unmarked individuals were captured, marked if needed, and sampled for pollen if present. The entrance tube was cleaned between sampling sessions but not between bumblebee individuals. We assume the level of contamination of pollen between individuals to be low, because the sampled pollen was concentrated in dense pellets on the bumblebees’ corbicula, each containing a huge number of pollen grains. We also assume contamination within individuals between foraging trips to be negligible. Later in the season, the work was focused on recapturing marked bumblebees, however, new bumblebees were also marked if pollen could be collected from them. Each monitored colony was visited at least once a week, unless it rained, or wind speed was > 6 m/s. Each monitoring session lasted for about 1 h, and all three colonies within the same area were visited on the same day. The time of sampling was between 9:00 and 18:00. The colonies were visited between the 8th of May and the 27th of June, but at the end of this period very few or no bumblebees could be sampled because the colonies were at the end of their cycles. The total sampling effort of pollen from bumblebee individuals in each of the colonies can be inferred from Appendix B of the Supplementary material.

### Pollen analysis

The pollen loads from individually sampled bumblebees (both, or sometimes only one of the pollen pellets whenever the other had been lost during handling) were stored in Eppendorf tubes in a −18°C freezer, and subsequently in an 8°C fridge after 70% ethanol had been added to the samples. Each sample diluted in ethanol was homogenized with a vortex mixer just before a small amount of the suspension was transferred with a pipette to an approximately 3 mm^3^ cube of fuchsin-stained gelatin gel on a microscope slide. The gel cube with the pollen dilution on top was instantly melted on a heat plate and thinned out by gently pressing a cover slide on top. Subsequently, the identity and relative frequency of pollen “types” (the lowest possible taxa for reliable identification, in some cases species, and others genus or family, Appendix C of the Supplementary material) were examined with an automated method for pollen analysis described in Olsson et al. (2021). In short, the pollen sample slides were scanned into high-resolution (0.25 µm) images, and then a pre-trained deep learning convolutional neural network (CNN) model classified each pollen grain. The CNN is trained using a reference library with pollen from over plant 200 species. Each reference sample is collected from the flower (or flowers) of an individual plant in the field, that has been identified with certainty, by gently rubbing a fuchsine gel cube against the flower’s anthers. For the current analysis, we used 117 species, that all were represented by at least two reference samples and images of at least 1000 individual pollen grains. The species were grouped into 36 pollen-type classes, consisting of morphologically similar pollens that are hard to separate—both for humans and the CNN. All species and groups are listed in Appendix C of the Supplementary material. The accuracy of the CNN, when classifying to groups as we do here, has been shown to be 80–90% (Olsson et al. 2021). The CNN works by classifying each pollen grain, giving a probability of identity of the different pollen types. These probabilities were summed for each sample, and the resulting frequencies per pollen type were adjusted according to Olsson et al. (2021) to reduce the effects of contamination and uncertain classifications. Each analyzed sample constitutes the pollen content of the pollen baskets of an individual bumblebee at a given time point and is what we mean when we henceforth refer to “pollen samples.”

### Flower constancy and diversity measurements

To address flower constancy during single foraging trips, we used a threshold value of 95% of a single pollen type in a pollen sample to separate between constant and in-constant pollen samples (following previous studies, Leonhardt and Blüthgen 2012; Somme et al. 2015). This approach reflects a stricter definition of flower constancy restricted to a single species preference. To address whether pollen foraging preferences linger over time, we assessed the similarity between all pollen samples by creating quantitative Jaccard distances (Tuomisto 2010b) ranging from 0 to 1 between all the samples, with the “vegdist” function from the *vegan* package (Oksanen et al. 2019). These were inverted (1-distance) to produce similarities. Quantitative Jaccard distances measure the difference in composition between the “communities” of pollen species in two samples while considering both the pollen-type identities and their quantities. Thus, two samples that do not share any species will have a distance of 1 (similarity of 0), while if they share all species, but not in identical proportions, then distance will be close to 0 and the similarity close to 1. This approach reflects consistent foraging behaviors, regardless of the number of included species. To separate the two approaches, they are henceforth referred to as flower constancy, and pollen sample similarity, respectively. The reason why we did not use other established measures of flower constancy, such as Bateman’s constancy index (Bateman 1951) or Gegear and Thomson’s constancy index (Gegear and Thomson 2004), is that those require knowledge of movement sequences between flowers. Additionally, these indices focus on flower visits regardless of the kind of foraging (nectar or pollen foraging), while we only focus on pollen foraging in our measures.

We assessed the degree of specialization, or diet breadth, of individual bumblebee’s pollen loads by estimating the effective number of species of pollen in pollen samples as Shannon diversity (Hill 1973; Jost 2006), using the “renyi” function from the R package *vegan* (Oksanen et al. 2019). Similarly, we estimated the Shannon diversity of all pollen collected in a colony. The pollen diversity of individual pollen samples can be considered as α diversity and that of the colony as γ diversity. The true β diversity is generally calculated as γ/mean(α) (Tuomisto 2010a), and gives an estimate of variability among workers. To estimate β for our sample of colonies we regress γ on α, and provided the line goes through the origin the slope estimates β. To capture a snapshot in time for the γ diversity measurements, the data was divided into three different time periods, early (8–21 May), mid (22 May–6 June), and late (14–27 June). The time periods correspond to 2 weeks of fieldwork each, and the gap between the mid and the late time period was caused by bad weather conditions. These time periods were only used to analyze diversity relations, in all other analyses, we use time of season or time between samples as a covariate.

It should be noted that the coarseness of possible identification of certain species groups (such as many genera within Rosaceae, see Appendix C of the Supplementary material), makes it likely that our measurements of pollen diversity are slightly underestimated, and measurements of flower constancy and pollen sample similarity slightly overestimated.

### Statistical analyses

All data processing and statistical analyses were performed in R (version 4.0.3, RCoreTeam 2020), using the packages *stats* and *lme4* (Bates et al. 2015), see below for details. In cases where the effects of interactions between fixed factors were tested, they were removed if not significant based on *P*-values derived from type II Wald χ^2^ tests. A reduced model was then rerun and the final models only contained main factors and significant (*P* < 0.05) interactions. To verify that test assumptions were met, we visually inspected the full models’ residuals and tested for deviations from the normal (Kolmogorov–Smirnov test), dispersion, and outliers with the *DHARMa* package (Hartig 2018). Unless mentioned in connection to the test descriptions below, assumptions were met. For analyses including > 1 predictor, multicollinearity was checked with the “check_collinearity” function from the package *performance* (Lüdecke et al. 2020). VIF-values were < 5 in all cases. Graphs displaying model estimates were produced from values extracted with the R package *effects* (Fox and Weisberg 2019).

#### Flower constancy during single trips

The effect of time (across the sampling period) and distance to oilseed rape on flower constancy (binary response variable) was analyzed with a binomial GLMM (generalized linear mixed model) with function glmer in package *lme4* (Bates et al. 2015), with bee ID as a random grouping factor, using the bobyqa optimizer (package *optimx*, Nash and Varadhan 2011). A preliminary analysis showed that the variance explained by the colony ID and area was numerically zero, and hence was not included as random factors. As this model only contains between-subject factors, we did not include random slope terms. To explore the differences between oilseed rape distances, a Tukey post-hoc test was performed with the “glht” function from the R package *multcomp* (Hothorn et al. 2008).

#### Pollen sample similarity over time

Jaccard similarity between pollen samples was analyzed as a function of individual (whether compared samples were from the same individuals or not), colony (whether compared samples were from the same colonies or not), days between samples, and the interaction between “days between samples” and the two former variables, respectively, using a linear mixed model (LMM; function lmer in package *lme4*). The ID, and colony ID nested within area, of the first bumblebee in each similarity comparison was added as random grouping factors. Random slopes were included for both the “individual” factor, colony factor and days between samples, as these are within-subject factors. For this model, the Kolmogorov–Smirnov residual test was significant (*P* < 0.001), which may be a consequence of the large number of data points (in this case > 5000). As a visual inspection of a qqplot did not indicate any important deviations (Appendix D of the Supplementary material), we decided to keep the model. In addition, the outlier test was significant (*P* = 0.003); a graphical view of this is presented in Appendix D of the Supplementary material.

#### Pollen diversity

The impact of time of season, distance to oilseed rape and the interaction between these, on α diversity was tested with an LMM, with colony ID nested within area, and bumblebee ID as random factors. In this model, the response variable was square-root transformed after subtracting 1 (such that the distribution starts at 0 instead of 1, as 1 otherwise is the minimum value of a Hill number), to achieve linearity.

To assess the relationship between individual and nest pollen diversity for each of the time periods (early; 8–21 May, mid; 22 May–6 June, and late; 14–27 June), we performed linear models analyzing γ diversity as a function of α diversity (averaged by colony over the given time period).

## RESULTS

### Flower constancy

**Figure 2:**
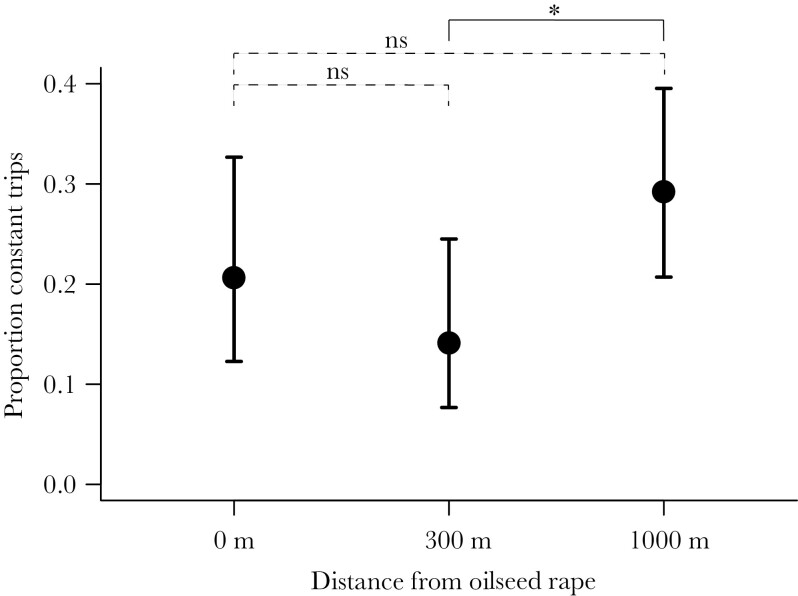
Proportion of pollen foraging trips that were constant, defined as consisting of >95% of a single pollen resource, at the three distances ~0, 300, and 1000 m from oilseed rape. Fitted means and 95% confidence intervals are shown.

The bumblebees showed strict flower constancy in 23% of all sampled pollen foraging trips. The degree of flower constancy did not change over the season (χ^2^ = 1.704, *df* = 1, *P* = 0.19), but did differ between bumblebees in colonies at different distances to oilseed rape (χ^2^ = 8.11, *df* = 2, *P* = 0.017, Figure 2), with constancy being higher at 1000 m compared to 300 m away from oilseed rape fields (*z* = 2.81, *P* = 0.014), but with no difference between 0 and 300 m (*z* = −1.25, *P* = 0.4), or between 0 and 1000 m (*z* = 1.49, *P* = 0.3).

**Figure 3:**
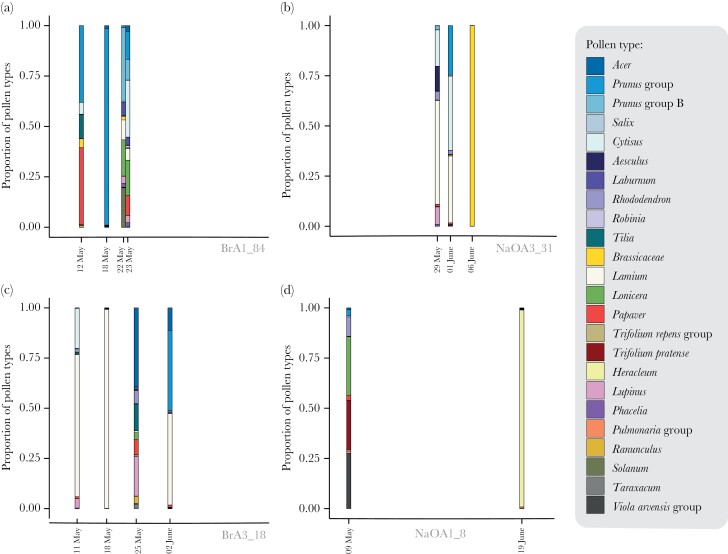
Pollen collected on different dates exemplified by four different bumblebee individuals: (a), (b), (c), and (d). Each bar represents a single pollen sample from each of the individuals.

Out of 300 sampled bumblebees 49 were caught more than once (Appendix A of the Supplementary material), and of these, four individuals were flower constant on the same pollen type at more than one occasion. One was captured twice on the same day showing constancy on *Heracleum* (which grew directly at the site), and three were constant on Brassicaceae with 3, 5, and 7 days between the sampling sessions for each respective individual. Additionally, one individual captured with 4 days in between showed strict flower constancy on two different taxa, *Salix* and Prunus group (Appendix C of the Supplementary material). Repeatedly sampled individuals showed many instances of a mix of constant and multiple species foraging trips, as well as complete pollen diet shifts over time (Figure 3, Appendix A of the Supplementary material). There were also many instances of individuals collecting multispecies pollen loads similar in composition with one or a few days between (e.g., Figure 3a, b).

**Figure 4:**
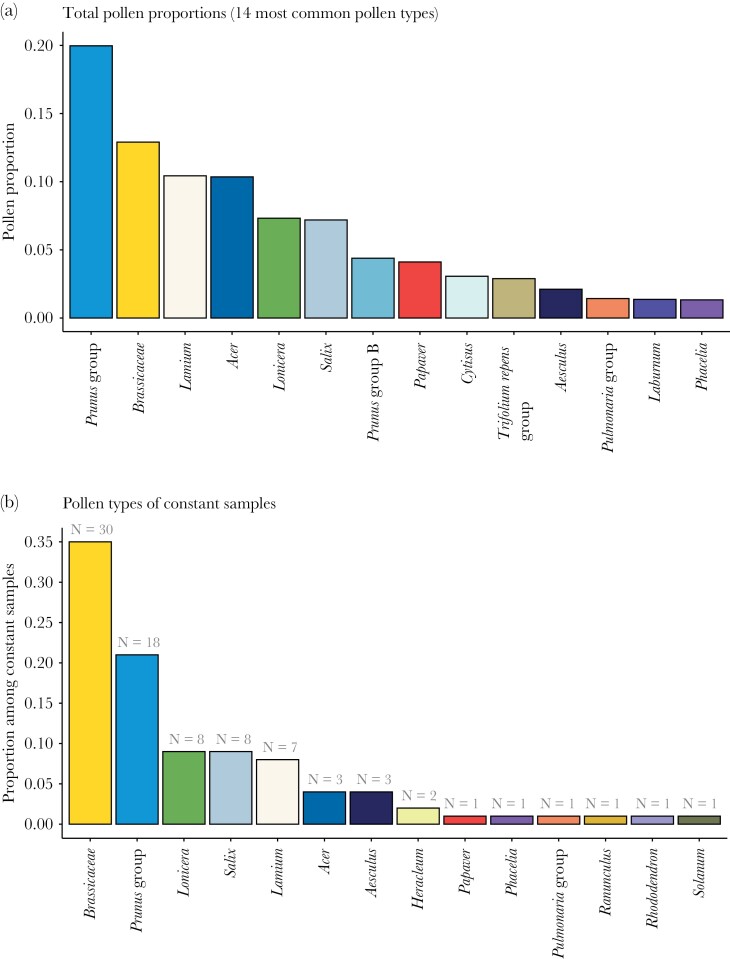
(a) Proportions of the 14 most common pollen types among all pollen samples, and (b) proportions and numbers of all constant pollen samples (samples with > 95% of a single pollen type), with proportion shown on the *y* axis and number of samples noted above each bar.

**Figure 5:**
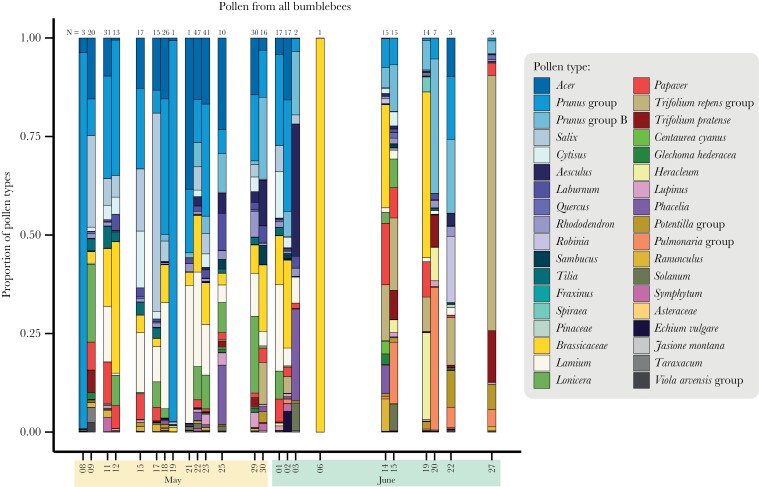
Pollen collected by all bumblebees, first averaged on colony, then on date. The shades of blue at the upper part of the bars represent woody plants. The number of individuals sampled at each date is noted above each bar.

Brassicaceae pollen was the most common pollen type among the flower constant pollen samples, making up 35% of the samples (Figure 4b), while pollens from the *Prunus* group (including *Prunus*, *Malus*, *Pyrus*, etc., see Appendix C of the Supplementary material) were the most common among all samples constituting 20% of all collected pollen, followed by Brassicaceae, *Lamium*, and *Acer* pollen (Figure 4a). The frequency of pollen types in all samples over time is shown in Figure 5.

### Pollen sample similarity

**Figure 6:**
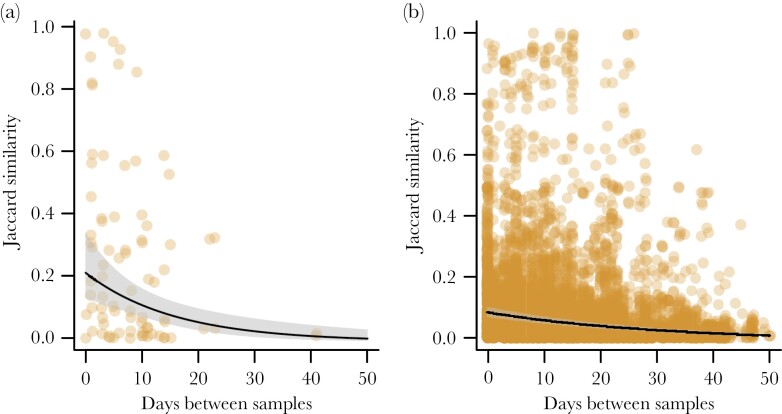
Quantitative Jaccard similarity between pollen samples within (a) and between (b) individual bumblebees, with time between samples on the *x* axis. Fitted means, 95% confidence intervals, and data points are shown.

Similarities between pollen samples declined with time between samples (χ^2^ = 352.2, *df* = 1, *P* < 0.0005), and samples from the same individual were more similar than samples from different individuals (χ^2^ = 9.74, *df* = 1, *P* = 0.002; Figure 6). The interaction between time between samples and the factor indicating same versus different individuals was not quite significant, (χ^2^ = 3.75, *df* = 1, *P* = 0.053), but we decided to let it remain in the model. Whether samples were from same or different colonies did not impact similarity between pollen samples (χ^2^ = 0.66, *df* = 1, *P* = 0.42).

### Pollen diversity

**Figure 7:**
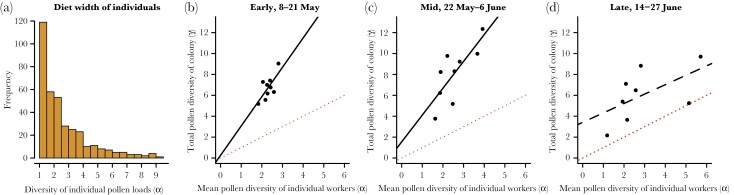
Histogram of α diversities in all pollen samples (a), and the diversity of pollen types collected by individuals (mean a per colony) plotted against the γ diversity of pollen per colony, during (b) the early (8–21 May), (c) mid (22 May–6 June), and (d) late (14–27 June) period of the colony lifetime. Fitted relations are shown as black lines, dashed in case of no significant relationship. The red, dashed lines showing a 1:1 relationship between γ and α diversity, are added for comparison.

The average diversity of pollen per foraging trip (α pollen diversity) was 2.54 ± 1.45 SD, ranging from 1.00 to 9.03 species per pollen sample (Figure 7a). Alpha (α) pollen diversity was not affected by the interaction between time of the season and oilseed rape distance (χ^2^ = 0.12, *df* = 2, *P* = 0.94). Nor did α pollen diversity change with time of the season (χ^2^ = 0.31, *df* = 1, *P* = 0.58), or with distance to oilseed rape (χ^2^ = 1.52, *df* = 2, *P* = 0.47) in a model without the interaction.

The pollen diversity of the colonies, that is, γ diversity, was positively related to that of individual foraging trips in the early (8–21 May; *F*_1,7_ = 7.040, *P* = 0.033, β = 2.81) and middle (22 May–6 June; *F*_1,7_ = 10.32, *P* = 0.015, β = 2.57) part of the nesting season, but not in the late part of the nesting season (14–27 June; *F*_1,6_ = 2.99, *P* = 0.13, β = 0.90, Figure 7). In the two first parts of the season regressions were significant, and the zero-intercept was not different from 0 (*P* = 0.9 and *P* = 0.5, respectively), and thus the slopes can be used as estimates of β diversity.

## DISCUSSION

In this study, we investigated different aspects of bumblebee pollen foraging over time. Contrary to expectations, we found that *B. terrestris* individuals displayed rather low levels of flower constancy during single trips, and conversely on average collected several types of pollen each, while the comparatively larger diversity collected at the colony level show that different individuals collect pollen from different flower species. We found no evidence for temporal variation in the level of flower constancy and the pollen diversity per foraging trip, but we could show that individuals were to some degree consistent in their pollen foraging choices over short temporal scales. We believe that our findings are important, not least because they are based on a field experiment, whereas the majority of previous results have been based on laboratory studies.

### Lower flower constancy than expected

We found that the workers of *B. terrestris* were flower constant (on pollen resources) in nearly a quarter of the single foraging trips throughout the study period, which is lower than the 33% and 76% shown in earlier (comparably small-scale) field studies on the same species (Leonhardt and Blüthgen 2012; Somme et al. 2015). The low number could be explained by the landscape potentially being resource poor, as our study area consisted of mainly large-scale agricultural land (cf. Persson and Smith 2013). Theory predicts a positive relationship between resource density (landscape quality) and degree of flower constancy (Hayes and Grüter 2022), regardless of the mechanistic reason behind flower constancy (Chittka et al. 1999; Grüter and Ratnieks 2011). That is, sufficient availability of good resources will allow specialization (Pulliam 1974), and with increased traveling between flowers the costs of sampling new flowers and learning, or retrieving memories, may be outweighed by travel costs (Waser 1986; Chittka et al. 1997). In our study, the colonies were placed at different distances from oilseed rape, a mass-flowering crop that may be used by *B. terrestris*, for nectar as well as for pollen (Westphal et al. 2009; Rundlöf et al. 2015). With these added flower resources, a higher degree of flower constancy could thus be expected, but when controlling for the oilseed rape distance we instead saw higher flower constancy at the largest distance from oilseed rape (but only in comparison to the medium distance, Figure 2). As the replication of the oilseed rape contrast is low (three colonies per distance) we do not want to draw any conclusions from this, somewhat puzzling, result. Oilseed rape was not widely used as a pollen resource (pollen from the plant family Brassicaceae, which includes oilseed rape and other present species, contributes to 13% of the pollen collected by all individuals, Figure 4a) but was at the same time the most common taxon in constant pollen samples (Figure 4b). This might be because whenever encountered, an oilseed rape field provides plenty of resources of one kind, but little of other kinds.

The few bumblebee individuals we saw that were flower constant on the same pollen type twice were all caught within a maximum of 7 days, and many individuals went from being flower constant to collecting a diversity of pollen within a few days (Appendix A of the Supplementary material). This indicates that flower constancy on certain species does not generally linger over longer time periods under natural conditions in *B. terrestris* (cf. Russell et al. 2017), even though the propensity to be flower constant may not change over time.

### Pollen sample similarity goes down over time

The similarity of pollen samples within and between individuals declined with time between pollen samples (Figure 6), which means that the pollen types they use do change over time (which can also be seen in Figure 2). This is likely explained by a change in the flowering species over the season, and the slightly steeper slope within individuals is explained by higher similarities between pollen samples from the same individuals over short time periods (Figure 6), indicating repetitive foraging behaviors. With the resolution of our data, we cannot determine how well and rapidly the bumblebees react to changing flower resources (although we can estimate that the similarity between pollen samples of the same individuals on average drops by 35% per week), but the change in bumblebee behavior may very well match the temporal change in availability of natural and farmed resources. This may not be surprising, as *B. terrestris* workers have been found to make exploratory foraging trips during 25% of the trips a day, even after having learned what resources are available (Lihoreau et al. 2010). Comparing Figure 6a and b shows that after approximately 2 weeks within-individual similarity has declined to the same level as a between-individual similarity. Furthermore, the similarity with any other individual today (0 days between samples) is approximately the same as the similarity between two samples from the same individual within a week or 10 days. Thus, after approximately that amount of time, individuals seem to have turned over their preferences.

### High α pollen diversity relates to even higher γ diversity

We found that the pollen diet width on single foraging trips (α diversity) remained the same across the season, with an average effective number of 2.54 species per trip. However, at the colony level (γ diversity) the diversity of pollen was nearly three times as large during the early and middle time period, and about twice as large in the late period (Figure 7). The ratio between the pollen diversity collected by the colony and the individuals estimates the (true) β diversity (Whittaker 1972; Tuomisto 2010a), and in community ecology that is taken as a measure of the effective number of different communities represented by the samples (Jost 2007). Here, we could think of it as the effective number of guilds of workers, each specializing in their group of flower species. In other words, the total variability among individual pollen samples in a colony is the same as if each colony had 2.7 (2.6–2.8) distinct classes of workers, with non-overlapping pollen diets consisting of 2.5 species. In reality, they are not distinct, of course, but the results show that individuals differ in what pollen species they collect.

Furthermore, the fact that between-sample similarity is greater within individuals than between, shows that individuals are often consistent in their choices, at least for some time. This partitioning could be driven by benefits to the individual of reductions in competition through spatial or flower-species niche separation, or be related to bees with different traits benefitting from specializing in different flowers (Johnson 1986; Peat et al. 2005). However, we acknowledge that with our data we cannot separate such active partitioning from a case where workers by chance encounter different rewarding species, and then stick to them. In addition, pollen diversity per se could benefit colony fitness, for example, by providing a balanced diet to larvae (Hass et al. 2018; Schweiger et al. 2022), but a mechanism for such foraging partitioning among bumblebee workers remains to be shown.

There are several reasons why individuals might collect a diversity of pollen types on single trips, and it is not possible to infer the exact ones without detailed information about the bumblebee individuals and their space use. Chittka et al. (1999) suggest that the costs of switching flowers when being familiar with a foraging area might decrease if bumblebees use spatial-contextual cues, or memories of sequences, facilitating the retrieval of handling memories of the different plant species, which may make diverse pollen foraging trips less costly. A diverse pollen foraging trip can also be the result of exploration trips, especially among inexperienced bumblebees (Osborne et al. 2013; Woodgate et al. 2016), but also among all bumblebees some levels of exploration are expected (Lihoreau et al. 2010; Kembro et al. 2019; Pasquaretta et al. 2019). Furthermore, the body size of bumblebee foragers impacts the foraging behavior: it has been shown that larger individuals are more efficient, selective, and flower constant when foraging (Klein et al. 2017; Frasnelli et al. 2020; Gervais et al. 2020), possibly due reduced relative flight costs and potentially increased sensory abilities with, for example, larger compound eyes. Another possible explanation is that plant species visited during the same foraging trip are morphologically similar so that the costs connected with changing focal flower resources are reduced (cf. Gegear and Laverty 2005), but visual inspection of our data does not support this idea.

### Conclusions and suggestions for future research

We conclude that flower constancy occurs in our population, but that it may be less common than found previously, and that the frequency of constant pollen foraging trips remains the same over the season. However, individual workers that were constant on a particular pollen type at one point often changed later to collect mixed pollen loads. Thus, constancy does not imply behavioral rigidity. Nevertheless, samples from the same individual are more similar than samples from different individuals, and we thus conclude that individuals show a degree of consistency in their pollen foraging over several days. To estimate how long this consistency remains, data with a higher temporal resolution for individuals, as well as available flower resources, are needed in future studies. Also, we call for additional research on inter- and intraspecific differences in the level, and temporal persistence, of flower constancy.

Like the level of constancy, the pollen diversity within samples did not change with season but remained rather constant with an average between two and three pollen types per sample. The much larger collective diversity of colonies than of individuals shows that different individuals focus on different flower species. However, we have not been able to assess if individuals’ foraging varies systematically in relation to, for example, their body size, or if colonies with higher pollen diversities have higher fitness. Another interesting focus of future studies would be to study how pollen diversity, and similarity among workers, is related to habitat quality and inter- and intraspecific competition.

## SUPPLEMENTARY MATERIAL

Supplementary material can be found at *Behavioral Ecology* online.

Supporting materials

**Appendix A** Pollen samples from all individuals sampled more than once.

**Appendix B** Aggregated pollen samples from each of the bumblebee nests over time.

**Appendix C** Species or species groups included in pollen identification analysis.

**Appendix D** Qqplot and plotted residuals displaying outliers from similarity model, produced by *DHARMa.*

arad028_suppl_Supplementary_Appendix_A

arad028_suppl_Supplementary_Appendix_B

arad028_suppl_Supplementary_Appendix_C

arad028_suppl_Supplementary_Appendix_D

## Data Availability

Analyses reported in this article can be reproduced using the data provided by Yourstone (2023).

## Notes

We want to thank and acknowledge Henrik Smith for initiating the over-arching project. We also thank the farmers and landowners for letting us put bumblebees on their land, Björn Klatt and Albin Belsing for assisting with the placement of the bumblebees and the sampling, Melanie Karlsson for guidance during laboratory work, and finally Charlie Nicholson, Henrik Smith, and three anonymous reviewers for providing constructive comments on the manuscript.
