# Supplementary material for: Bumblebee flower constancy and pollen diversity over time
Source: Behav Ecol. 2023 Apr 10;34(4):602–12. doi: 10.1093/beheco/arad028 (PMC10332455; doi:10.1093/beheco/arad028)
Supplement: arad028_suppl_Supplementary_Appendix_A [file arad028_suppl_supplementary_appendix_a.pdf]

Appendix A. Pollen loads and their content and sampling date from all individuals sampled more than once. The first part of the individuals' ID numbers (stated above each graph) reflects the study area, the second part the distance of the colony from oilseed rape (A1 = 0m, A2 = 300m, A3= 1000m), and the third part is a unique identifier.

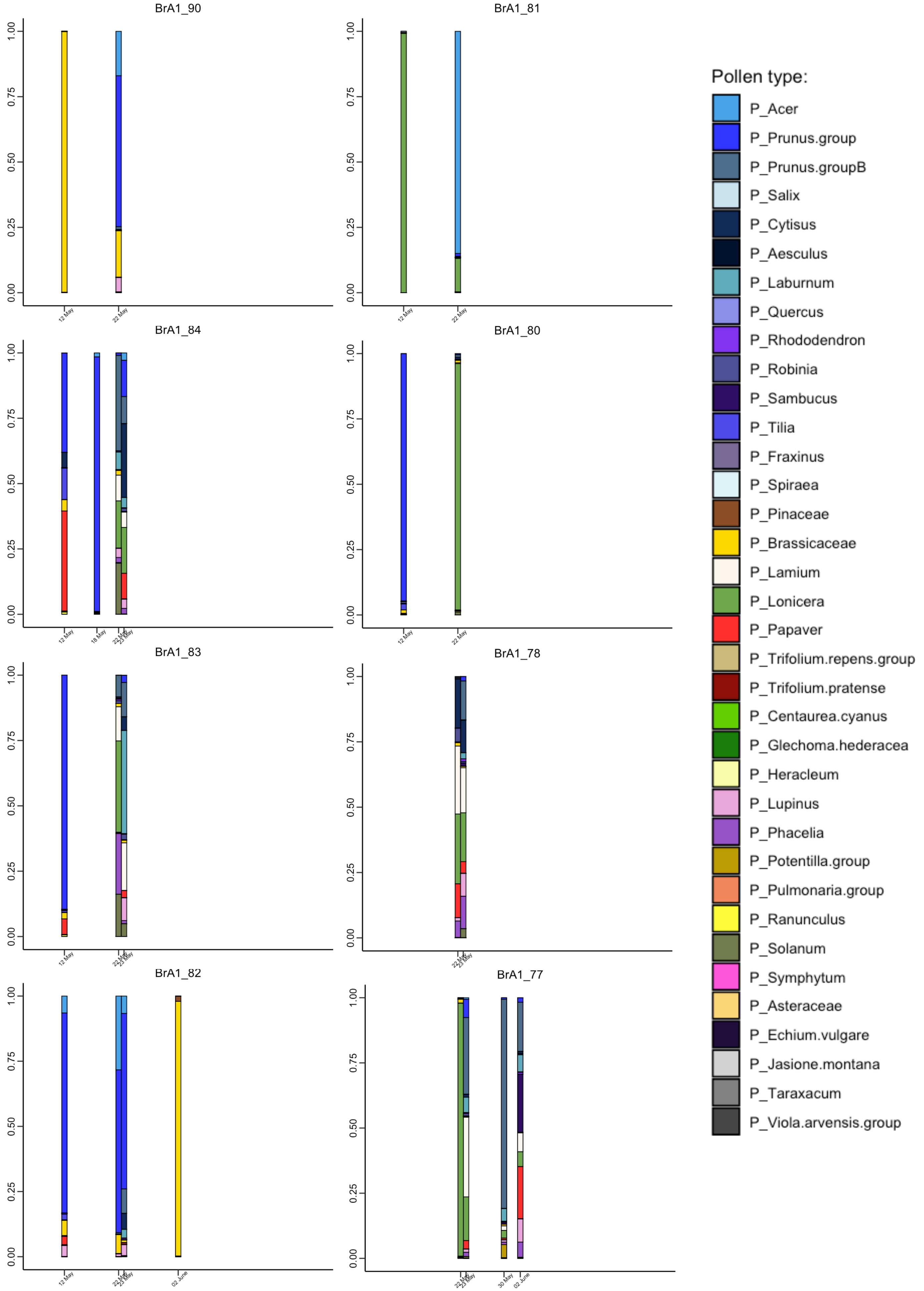

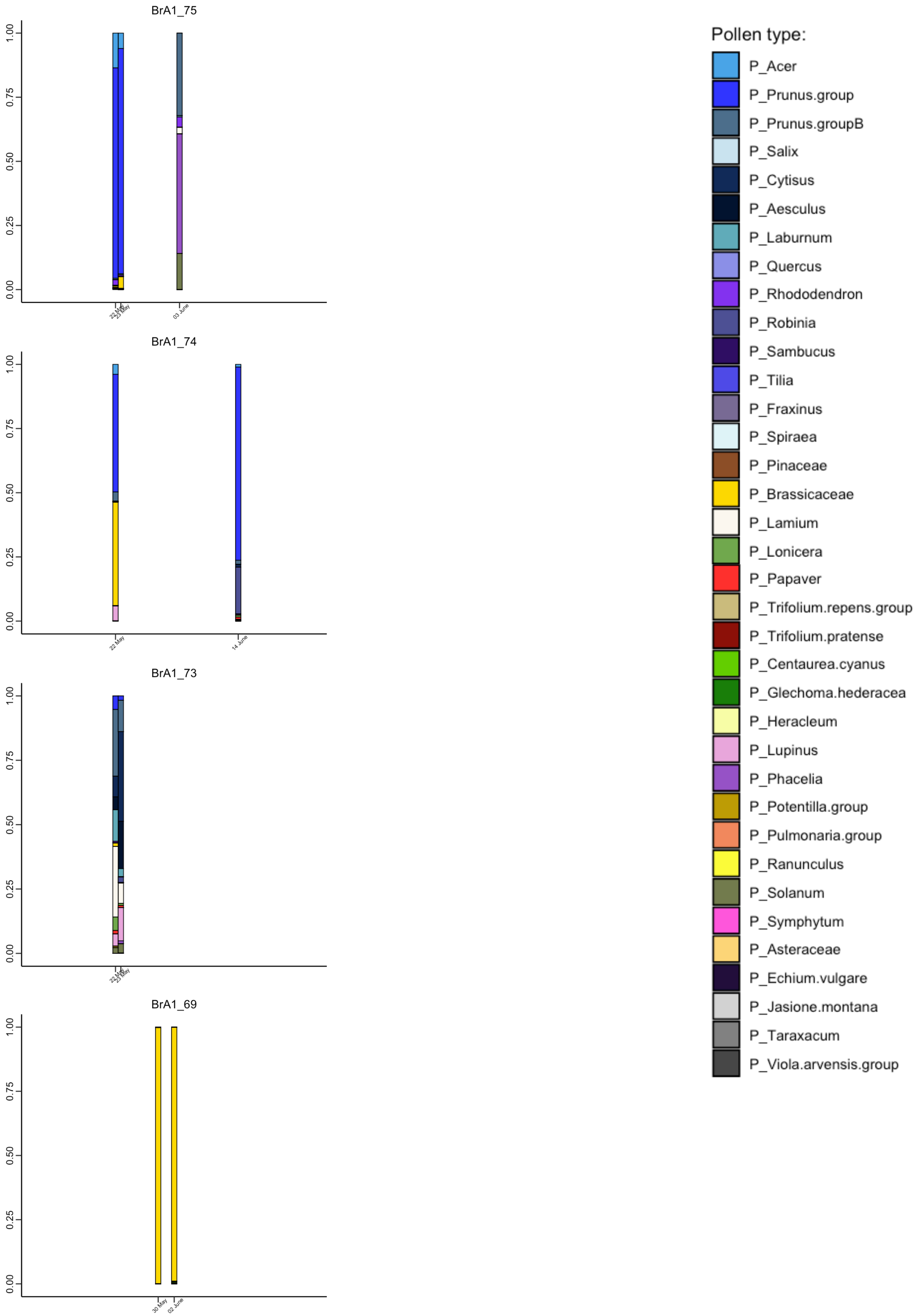
BrA1_75
1.00
0.75
0.50
0.25
0.00
22 May
23 May
03 June
BrA1_74
1.00
0.75
0.50
0.25
0.00
22 May
14 June
BrA1_73
1.00
0.75
0.50
0.25
0.00
22 May
23 May
BrA1_69
1.00
0.75
0.50
0.25
0.00
30 May
02 June
Pollen type:
P_Acer
P_Prunus.group
P_Prunus.groupB
P_Salix
P_Cytisus
P_Aesculus
P_Laburnum
P_Quercus
P_Rhododendron
P_Robinia
P_Sambucus
P_Tilia
P_Fraxinus
P_Spiraea
P_Pinaceae
P_Brassicaceae
P_Lamium
P_Lonicera
P_Papaver
P_Trifolium.repens.group
P_Trifolium.pratense
P_Centaurea.cyanus
P_Glechoma.hederacea
P_Heracleum
P_Lupinus
P_Phacelia
P_Potentilla.group
P_Pulmonaria.group
P_Ranunculus
P_Solanum
P_Symphytum
P_Asteraceae
P_Echium.vulgare
P_Jasione.montana
P_Taraxacum
P_Viola.arvensis.group

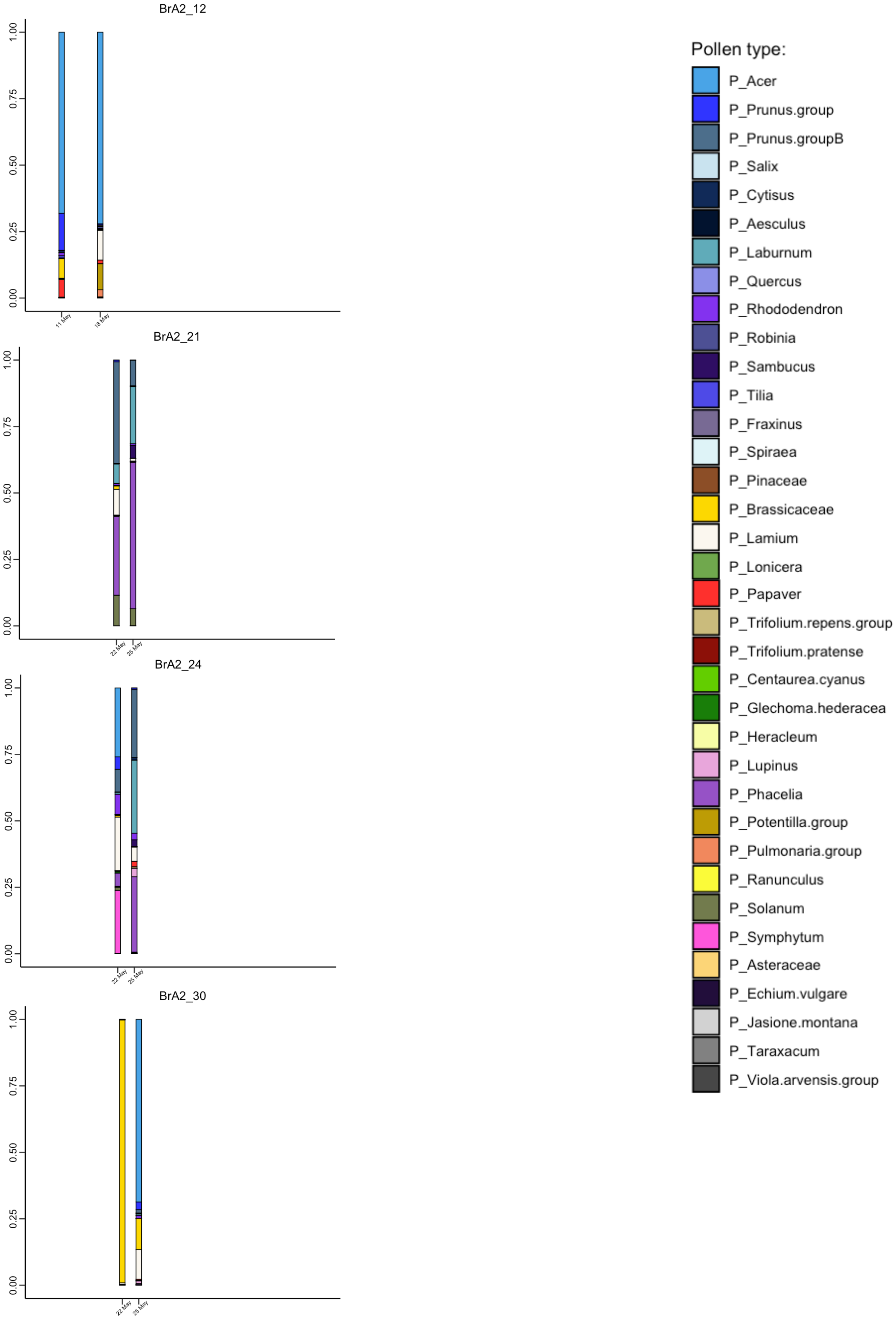
BrA2_12
1.00
0.75
0.50
0.25
0.00
11 May
18 May
BrA2_21
1.00
0.75
0.50
0.25
0.00
22 May
25 May
BrA2_24
1.00
0.75
0.50
0.25
0.00
22 May
25 May
BrA2_30
1.00
0.75
0.50
0.25
0.00
22 May
25 May
Pollen type:
P_Acer
P_Prunus.group
P_Prunus.groupB
P_Salix
P_Cytisus
P_Aesculus
P_Laburnum
P_Quercus
P_Rhododendron
P_Robinia
P_Sambucus
P_Tilia
P_Fraxinus
P_Spiraea
P_Pinaceae
P_Brassicaceae
P_Lamium
P_Lonicera
P_Papaver
P_Trifolium.repens.group
P_Trifolium.pratense
P_Centaurea.cyanus
P_Glechoma.hederacea
P_Heracleum
P_Lupinus
P_Phacelia
P_Potentilla.group
P_Pulmonaria.group
P_Ranunculus
P_Solanum
P_Symphytum
P_Asteraceae
P_Echium.vulgare
P_Jasione.montana
P_Taraxacum
P_Viola.arvensis.group

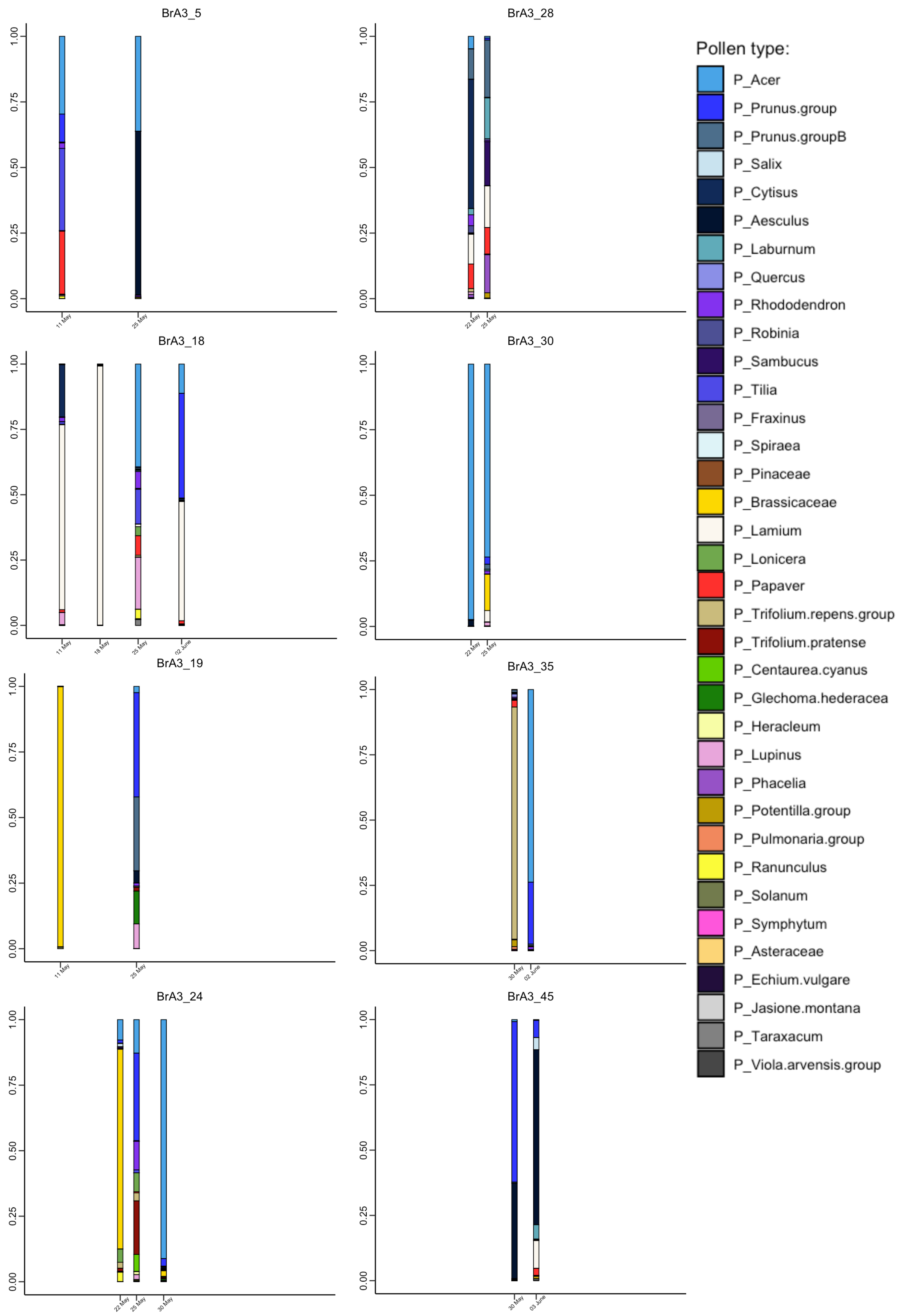

BrA3_5
11 May
25 May
BrA3_28
22 May
25 May
BrA3_18
11 May
18 May
25 May
02 June
BrA3_30
22 May
25 May
BrA3_19
11 May
25 May
BrA3_35
30 May
02 June
BrA3_24
22 May
25 May
30 May
BrA3_45
30 May
03 June
0.00
0.25
0.50
0.75
1.00
Pollen type:
P_Acer
P_Prunus.group
P_Prunus.groupB
P_Salix
P_Cytisus
P_Aesculus
P_Laburnum
P_Quercus
P_Rhododendron
P_Robinia
P_Sambucus
P_Tilia
P_Fraxinus
P_Spiraea
P_Pinaceae
P_Brassicaceae
P_Lamium
P_Lonicera
P_Papaver
P_Trifolium.repens.group
P_Trifolium.pratense
P_Centaurea.cyanus
P_Glechoma.hederacea
P_Heracleum
P_Lupinus
P_Phacelia
P_Potentilla.group
P_Pulmonaria.group
P_Ranunculus
P_Solanum
P_Symphytum
P_Asteraceae
P_Echium.vulgare
P_Jasione.montana
P_Taraxacum
P_Viola.arvensis.group

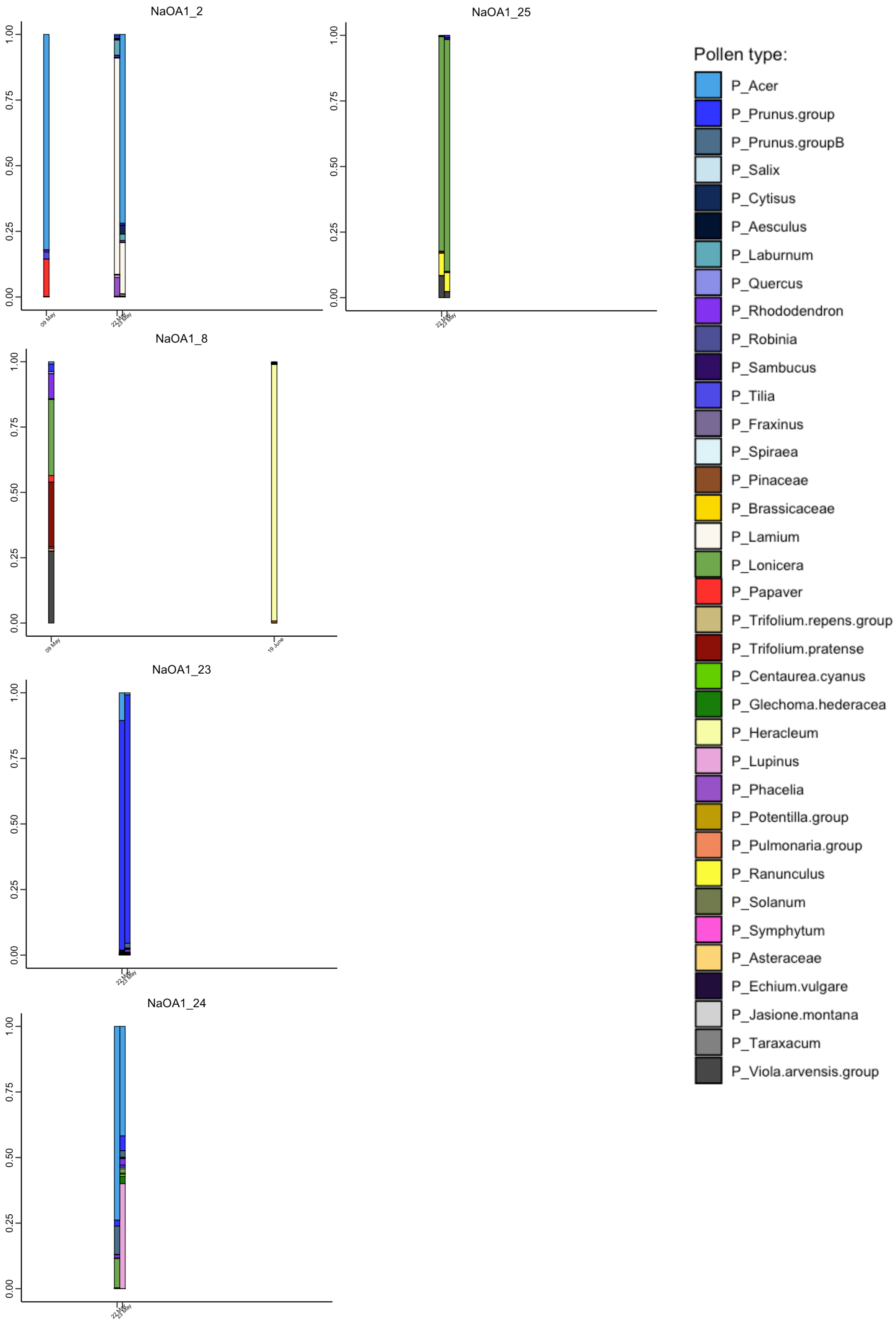
NaOA1_2
NaOA1_25
NaOA1_8
NaOA1_23
NaOA1_24
1.00
0.75
0.50
0.25
0.00
09 May
22 May
23 May
19 June
Pollen type:
P_Acer
P_Prunus.group
P_Prunus.groupB
P_Salix
P_Cytisus
P_Aesculus
P_Laburnum
P_Quercus
P_Rhododendron
P_Robinia
P_Sambucus
P_Tilia
P_Fraxinus
P_Spiraea
P_Pinaceae
P_Brassicaceae
P_Lamium
P_Lonicera
P_Papaver
P_Trifolium.repens.group
P_Trifolium.pratense
P_Centaurea.cyanus
P_Glechoma.hederacea
P_Heracleum
P_Lupinus
P_Phacelia
P_Potentilla.group
P_Pulmonaria.group
P_Ranunculus
P_Solanum
P_Symphytum
P_Asteraceae
P_Echium.vulgare
P_Jasione.montana
P_Taraxacum
P_Viola.arvensis.group

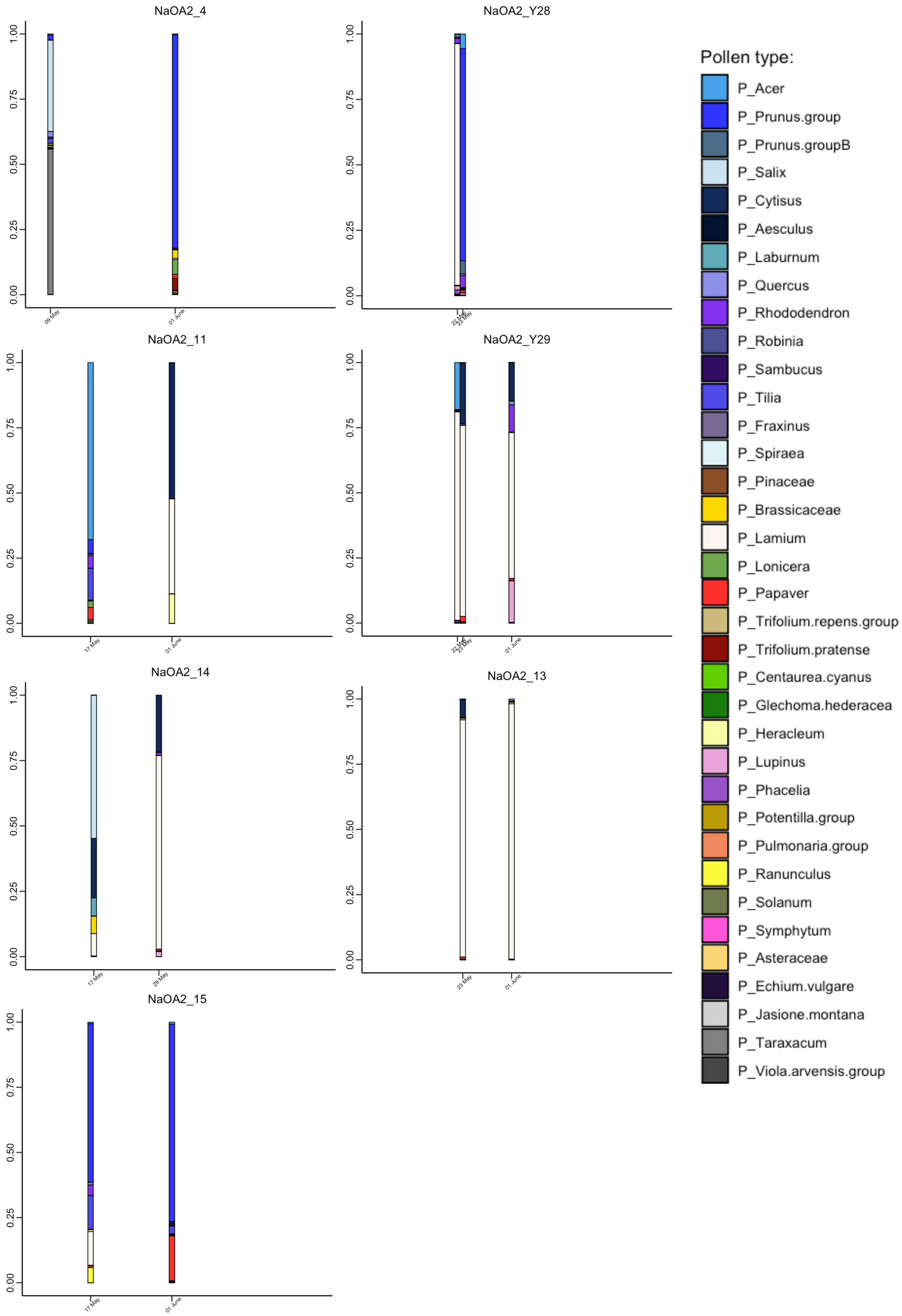
NaOA2_4
NaOA2_Y28
NaOA2_11
NaOA2_Y29
NaOA2_14
NaOA2_13
NaOA2_15
0.00
0.25
0.50
0.75
1.00
09 May
01 June
22 May
23 May
17 May
29 May
Pollen type:
P_Acer
P_Prunus.group
P_Prunus.groupB
P_Salix
P_Cytisus
P_Aesculus
P_Laburnum
P_Quercus
P_Rhododendron
P_Robinia
P_Sambucus
P_Tilia
P_Fraxinus
P_Spiraea
P_Pinaceae
P_Brassicaceae
P_Lamium
P_Lonicera
P_Papaver
P_Trifolium.repens.group
P_Trifolium.pratense
P_Centaurea.cyanus
P_Glechoma.hederacea
P_Heracleum
P_Lupinus
P_Phacelia
P_Potentilla.group
P_Pulmonaria.group
P_Ranunculus
P_Solanum
P_Symphytum
P_Asteraceae
P_Echium.vulgare
P_Jasione.montana
P_Taraxacum
P_Viola.arvensis.group

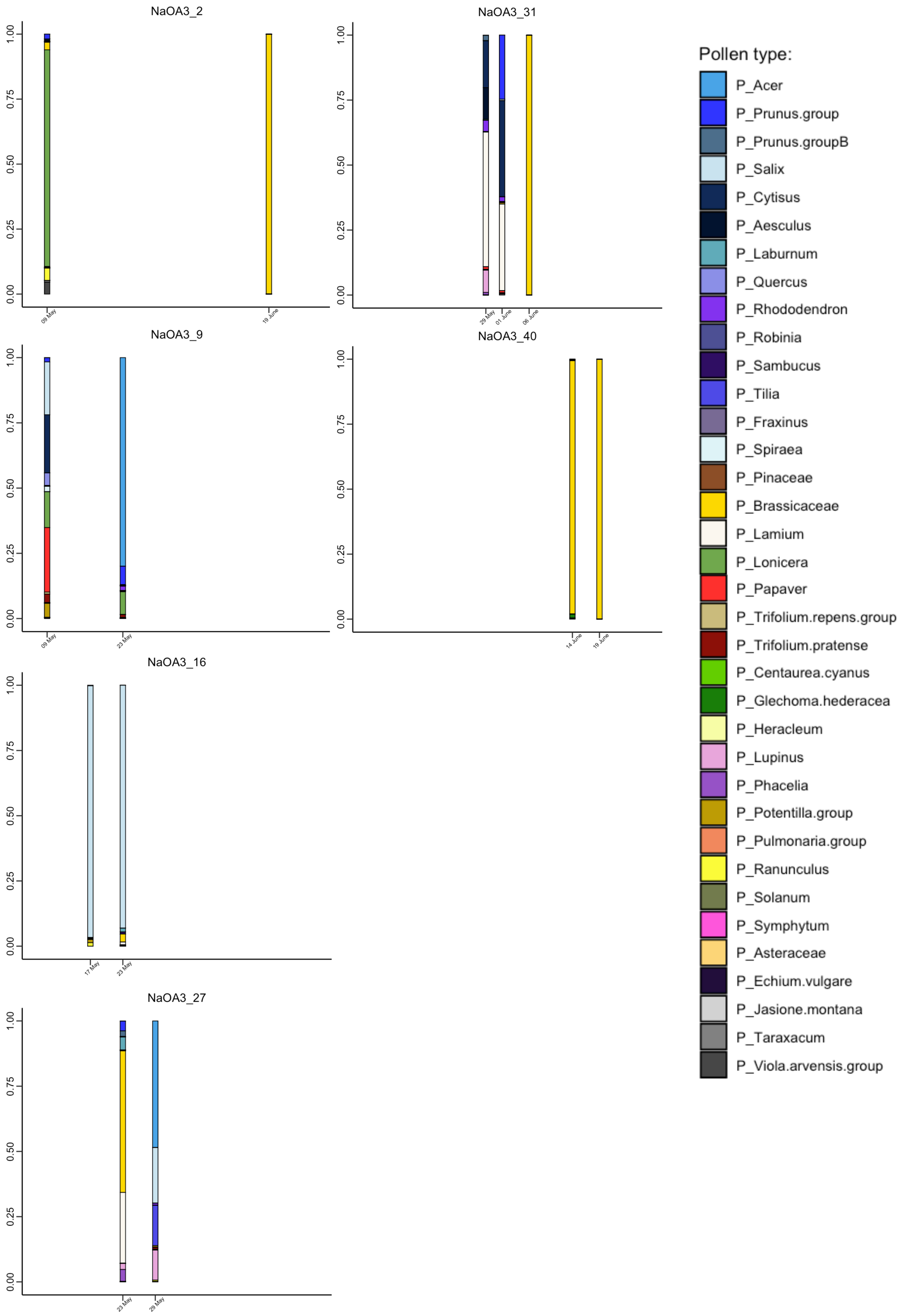

NaOA3_2
09 May
19 June
NaOA3_31
29 May
01 June
06 June
NaOA3_9
09 May
23 May
NaOA3_40
14 June
19 June
NaOA3_16
17 May
23 May
NaOA3_27
23 May
29 May
0.00
0.25
0.50
0.75
1.00
Pollen type:
P_Acer
P_Prunus.group
P_Prunus.groupB
P_Salix
P_Cytisus
P_Aesculus
P_Laburnum
P_Quercus
P_Rhododendron
P_Robinia
P_Sambucus
P_Tilia
P_Fraxinus
P_Spiraea
P_Pinaceae
P_Brassicaceae
P_Lamium
P_Lonicera
P_Papaver
P_Trifolium.repens.group
P_Trifolium.pratense
P_Centaurea.cyanus
P_Glechoma.hederacea
P_Heracleum
P_Lupinus
P_Phacelia
P_Potentilla.group
P_Pulmonaria.group
P_Ranunculus
P_Solanum
P_Symphytum
P_Asteraceae
P_Echium.vulgare
P_Jasione.montana
P_Taraxacum
P_Viola.arvensis.group

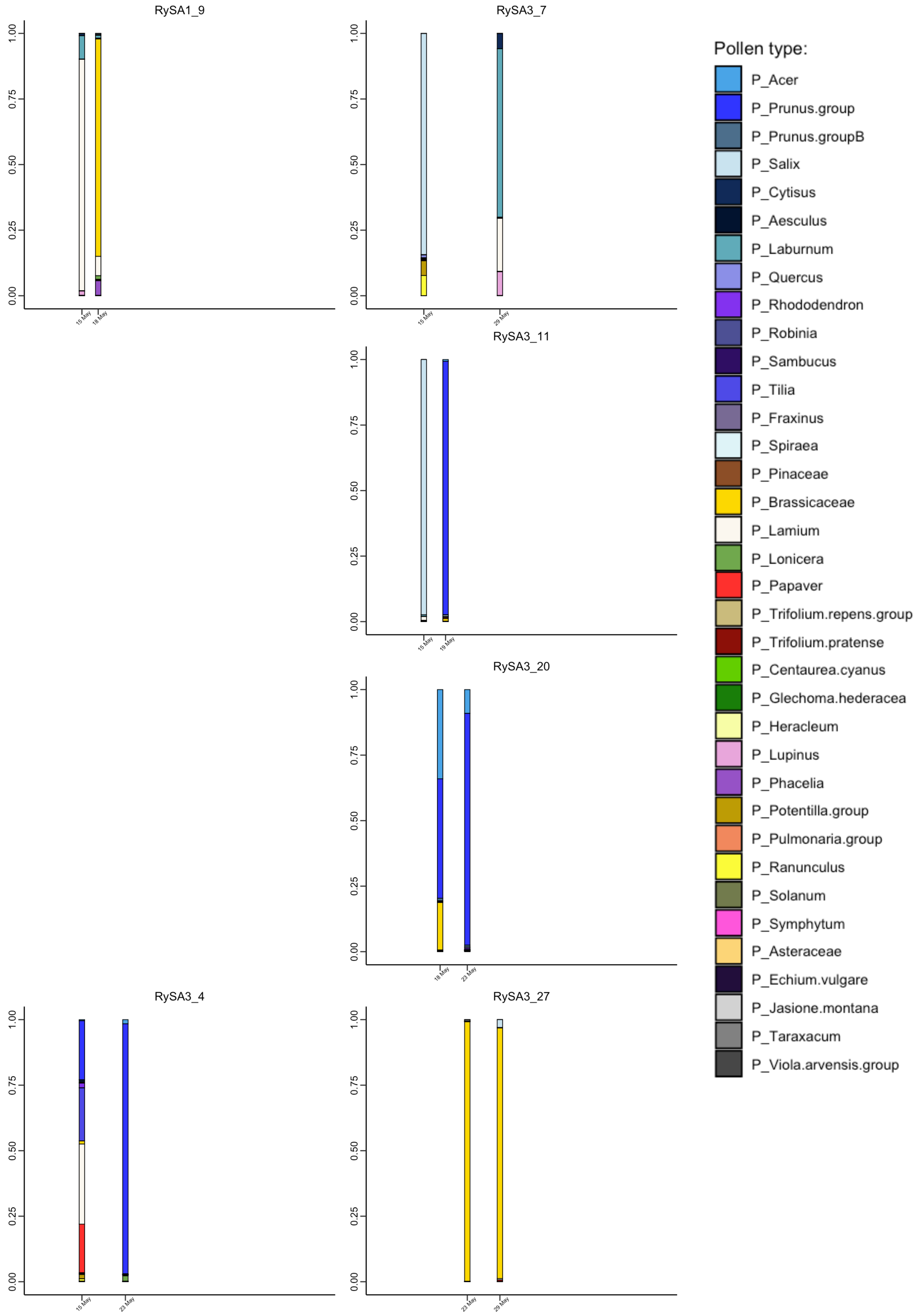

RySA1_9
RySA3_7
RySA3_11
RySA3_20
RySA3_4
RySA3_27
0.00
0.25
0.50
0.75
1.00
15 May
18 May
19 May
23 May
29 May
Pollen type:
P_Acer
P_Prunus.group
P_Prunus.groupB
P_Salix
P_Cytisus
P_Aesculus
P_Laburnum
P_Quercus
P_Rhododendron
P_Robinia
P_Sambucus
P_Tilia
P_Fraxinus
P_Spiraea
P_Pinaceae
P_Brassicaceae
P_Lamium
P_Lonicera
P_Papaver
P_Trifolium.repens.group
P_Trifolium.pratense
P_Centaurea.cyanus
P_Glechoma.hederacea
P_Heracleum
P_Lupinus
P_Phacelia
P_Potentilla.group
P_Pulmonaria.group
P_Ranunculus
P_Solanum
P_Symphytum
P_Asteraceae
P_Echium.vulgare
P_Jasione.montana
P_Taraxacum
P_Viola.arvensis.group
