# Supplementary material for: Bumblebee flower constancy and pollen diversity over time
Source: Behav Ecol. 2023 Apr 10;34(4):602–12. doi: 10.1093/beheco/arad028 (PMC10332455; doi:10.1093/beheco/arad028)
Supplement: arad028_suppl_Supplementary_Appendix_B [file arad028_suppl_supplementary_appendix_b.pdf]

Appendix B. All sampled pollen from each of the colonies over time, with different pollen types represented by different colors. Each bar represents a single sampling date, and the number above each bar shows the number of samples from that date. The first part of the colonies' ID numbers (stated above each graph) reflects the study area, and the second part the distance of the colony from oilseed rape (A1 = 0m, A2 = 300m, A3= 1000m).

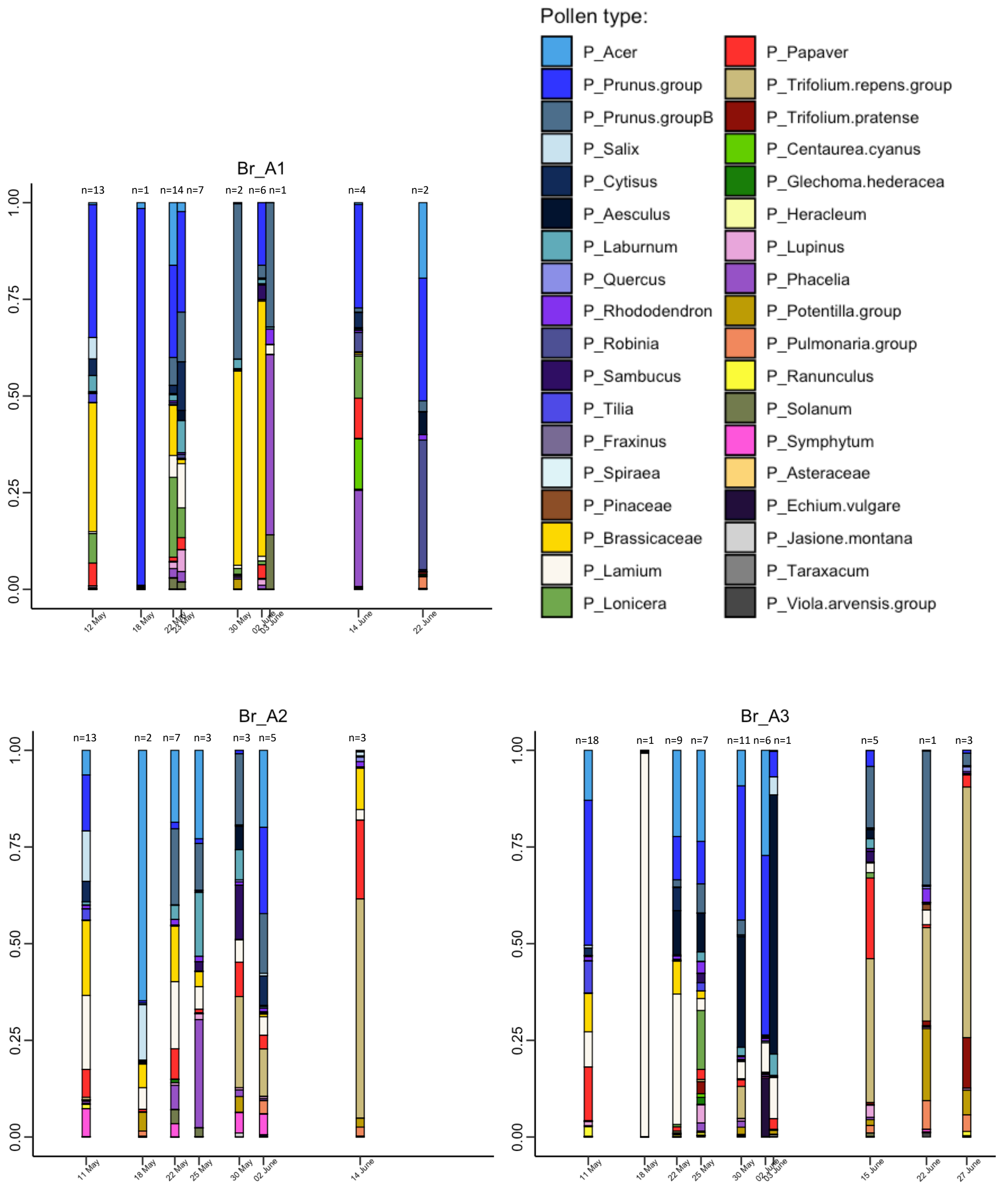

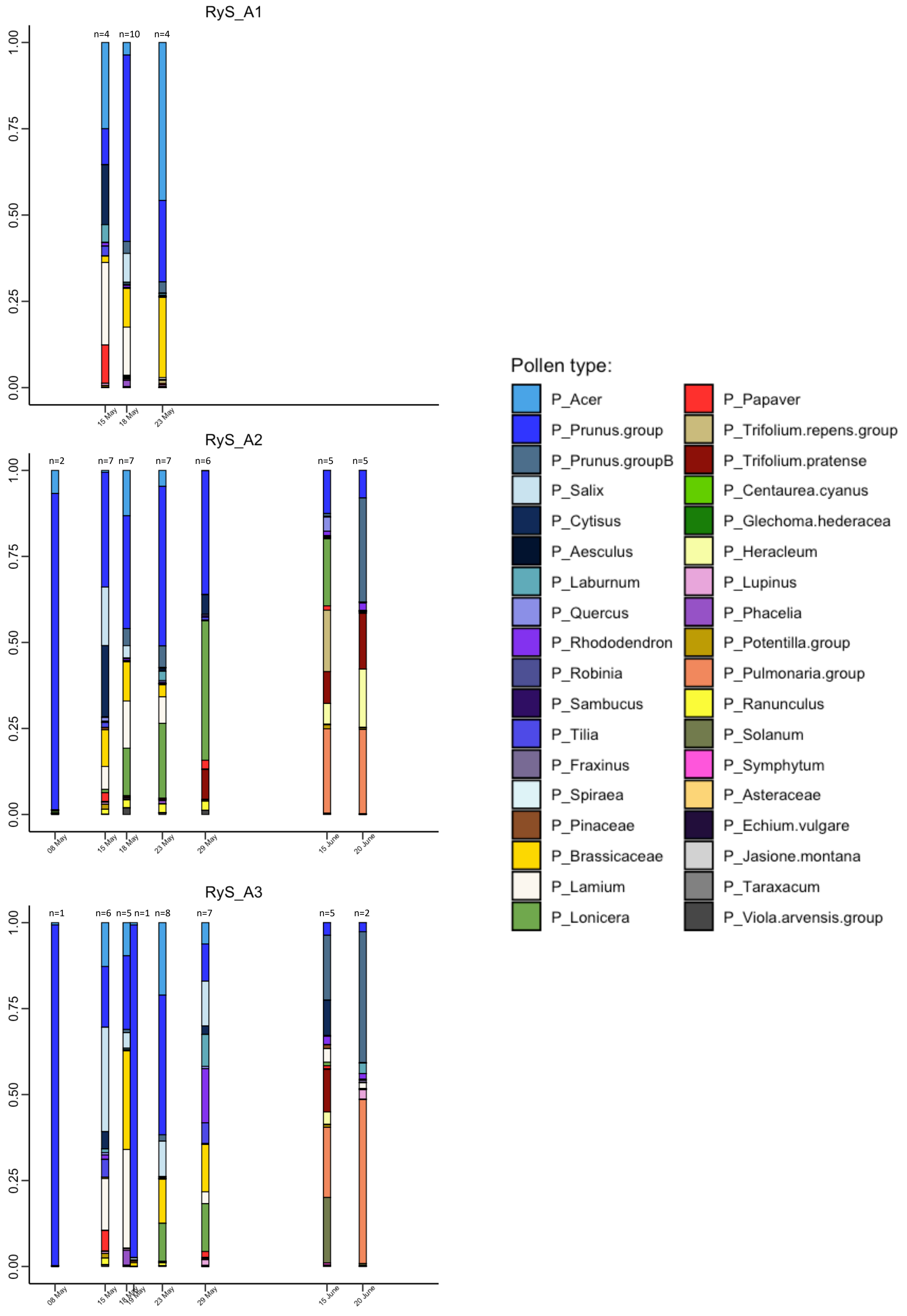
RyS_A1
n=4 n=10 n=4
1.00
0.75
0.50
0.25
0.00
15 May
18 May
23 May
RyS_A2
n=2 n=7 n=7 n=7 n=6 n=5 n=5
08 May
15 May
18 May
23 May
29 May
15 June
20 June
RyS_A3
n=1 n=6 n=5 n=1 n=8 n=7 n=5 n=2
08 May
15 May
18 May
19 May
23 May
29 May
15 June
20 June
Pollen type:
P_Acer
P_Prunus.group
P_Prunus.groupB
P_Salix
P_Cytisus
P_Aesculus
P_Laburnum
P_Quercus
P_Rhododendron
P_Robinia
P_Sambucus
P_Tilia
P_Fraxinus
P_Spiraea
P_Pinaceae
P_Brassicaceae
P_Lamium
P_Lonicera
P_Papaver
P_Trifolium.repens.group
P_Trifolium.pratense
P_Centaurea.cyanus
P_Glechoma.hederacea
P_Heracleum
P_Lupinus
P_Phacelia
P_Potentilla.group
P_Pulmonaria.group
P_Ranunculus
P_Solanum
P_Symphytum
P_Asteraceae
P_Echium.vulgare
P_Jasione.montana
P_Taraxacum
P_Viola.arvensis.group

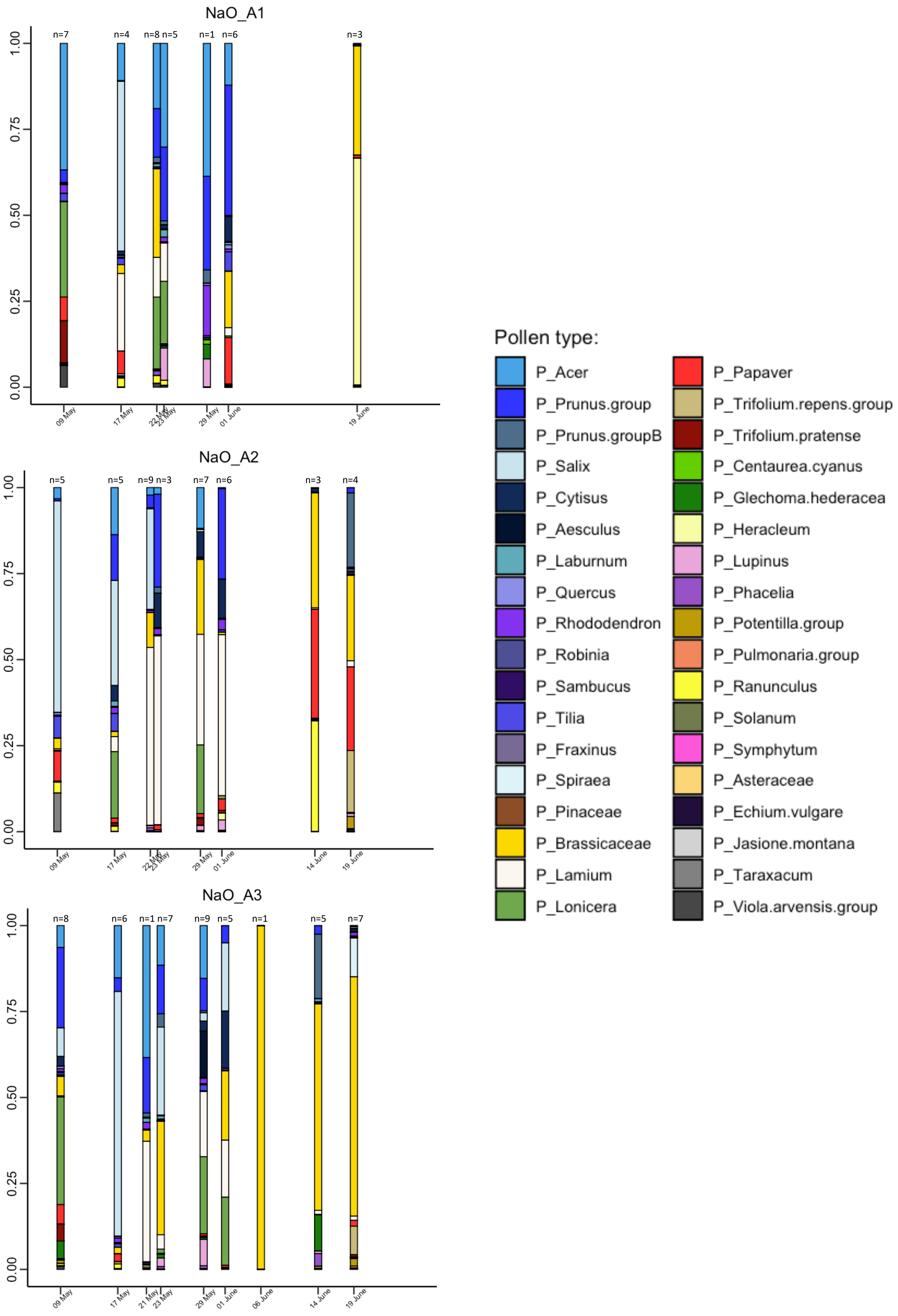
NaO_A1
n=7
n=4
n=8
n=5
n=1
n=6
n=3
1.00
0.75
0.50
0.25
0.00
09 May
17 May
22 May
23 May
29 May
01 June
19 June
NaO_A2
n=5
n=5
n=9
n=3
n=7
n=6
n=3
n=4
1.00
0.75
0.50
0.25
0.00
09 May
17 May
22 May
23 May
29 May
01 June
14 June
19 June
NaO_A3
n=8
n=6
n=1
n=7
n=9
n=5
n=1
n=5
n=7
1.00
0.75
0.50
0.25
0.00
09 May
17 May
21 May
23 May
29 May
01 June
06 June
14 June
19 June
Pollen type:
P_Acer
P_Prunus.group
P_Prunus.groupB
P_Salix
P_Cytisus
P_Aesculus
P_Laburnum
P_Quercus
P_Rhododendron
P_Robinia
P_Sambucus
P_Tilia
P_Fraxinus
P_Spiraea
P_Pinaceae
P_Brassicaceae
P_Lamium
P_Lonicera
P_Papaver
P_Trifolium.repens.group
P_Trifolium.pratense
P_Centaurea.cyanus
P_Glechoma.hederacea
P_Heracleum
P_Lupinus
P_Phacelia
P_Potentilla.group
P_Pulmonaria.group
P_Ranunculus
P_Solanum
P_Symphytum
P_Asteraceae
P_Echium.vulgare
P_Jasione.montana
P_Taraxacum
P_Viola.arvensis.group
