# Supplementary material for: Bumblebee flower constancy and pollen diversity over time
Source: Behav Ecol. 2023 Apr 10;34(4):602–12. doi: 10.1093/beheco/arad028 (PMC10332455; doi:10.1093/beheco/arad028)
Supplement: arad028_suppl_Supplementary_Appendix_C [file arad028_suppl_supplementary_appendix_c.pdf]

Appendix C. Table with the species or species groups that were identified with the CNN identification model, and the species that the model was trained on.

Pollen species/species group	*Species used in model*	Family
Acer	*Acer campestre*	Sapindaceae
	Acer platanoides	Sapindaceae
	Acer pseudoplatanus	Sapindaceae
Aesculus	*Aesculus hippocastanum*	Sapindaceae
Asteraceae	*Achillea millefolium*	Asteraceae
	Arctium tomentosum	Asteraceae
	Carduus crispus	Asteraceae
	Cichorium intybus	Asteraceae
	Cirsium arvense	Asteraceae
	Cirsium vulgare	Asteraceae
	Leucanthemum vulgare	Asteraceae
	Tripleurospermum inodorum	Asteraceae
Brassicaceae	*Alliaria petiolata*	Brassicaceae
	Barbarea vulgaris	Brassicaceae
	Brassica napus	Brassicaceae
	Capsella bursa-pastoris	Brassicaceae
	Cardamine pratensis	Brassicaceae
	Hesperis matronalis	Brassicaceae
	Thlaspi arvense	Brassicaceae
Centaurea cyanus	*Centaurea cyanus*	Asteraceae
Cytisus	*Cytisus scoparius*	Fabaceae
Echium vulgare	*Echium vulgare*	Boraginaceae
Fraxinus	*Fraxinus excelsior*	Oleaceae
Glechoma hederacea	*Glechoma hederacea*	Lamiaceae
Heracleum	*Heracleum sp*	Apiaceae
Jasione montana	*Jasione montana*	Campanulaceae
Laburnum	*Laburnum sp*	Fabaceae
Lamium	*Lamiastrum galeobdolon*	Lamiaceae
	Lamium album	Lamiaceae
	Lamium hybridum	Lamiaceae
	Lamium purpureum	Lamiaceae
Lonicera	*Knautia arvensis*	Caprifoliaceae
	Kolkwitzia amabilis	Caprifoliaceae
	Lonicera caprifolium	Caprifoliaceae
	Lonicera periclymenum	Caprifoliaceae
	Lonicera tatarica	Caprifoliaceae
	Lonicera xylosteum	Caprifoliaceae
	Valeriana dioica	Caprifoliaceae
Lupinus	*Lupinus polyphyllus*	Fabaceae
Papaver	*Chelidonium majus*	Papaveraceae
	Papaver dubium	Papaveraceae
	Papaver rhoeas	Papaveraceae
Phacelia	*Phacelia tanacetifolia*	Boraginaceae
Pinaceae	*Picea abies*	Pinaceae
	Pinus sylvestris	Pinaceae
Potentilla-group	*Fragaria vesca*	Rosaceae
	Geum rivale	Rosaceae
	Geum urbanum	Rosaceae
	Argentina anserina	Rosaceae
	Potentilla anserina	Rosaceae
	Potentilla argentea	Rosaceae
	Potentilla erecta	Rosaceae

	Potentilla fruticosa	Rosaceae
	Potentilla reptans	Rosaceae
Prunus-group	*Amelanchier confusa*	Rosaceae
	Amelanchier spicata	Rosaceae
	Cotoneaster bullatus	Rosaceae
	Cotoneaster divaricatus	Rosaceae
	Cotoneaster sp	Rosaceae
	Crataegus laevigata	Rosaceae
	Crataegus monogyna	Rosaceae
	Malus domestica	Rosaceae
	Prunus avium	Rosaceae
	Prunus domestica	Rosaceae
	Prunus laurocerasus	Rosaceae
	Prunus padus	Rosaceae
	Prunus spinosa	Rosaceae
	Pyrus communis	Rosaceae
	Sorbus aucuparia	Rosaceae
Prunus-groupB	*Ribes nigrum*	Grossulariaceae
	Ribes rubrum	Grossulariaceae
	Rosa helenae	Rosaceae
	Rosa multiflora	Rosaceae
	Rosa rugosa	Rosaceae
	Rubus fruticosus	Rosaceae
	Rubus idaeus	Rosaceae
Pulmonaria-group	*Anchusa officinalis*	Boraginaceae
Quercus	*Quercus robur*	Fagaceae
	Quercus rubra	Fagaceae
Ranunculus	*Ficaria verna*	Ranunculaceae
	Ranunculus acris	Ranunculaceae
	Ranunculus bulbosus	Ranunculaceae
	Ranunculus repens	Ranunculaceae
Rhododendron	*Rhododendron*	Ericaceae
Robinia	*Robinia pseudoacacia*	Fabaceae
Salix	*Salix alba*	Salicaceae
	Salix pil	Salicaceae
	Salix caprea	Salicaceae
	Salix cinerea	Salicaceae
	Salix euxina	Salicaceae
	Salix repens	Salicaceae
	Salix viminalis	Salicaceae
	Salix x fragilis	Salicaceae
Sambucus	*Sambucus nigra*	Adoxaceae
Symphytum	*Symphytum officinale*	Boraginaceae
	Symphytum x uplandicum	Boraginaceae
Taraxacum	*Bellis perennis*	Asteraceae
	Jacobaea vulgaris	Asteraceae
	Petasites hybridus	Asteraceae
	Pilosella officinarum	Asteraceae
	Senecio leucanthemifolius	Asteraceae
	Senecio vulgaris	Asteraceae
	Taraxacum	Asteraceae
	Tussilago farfara	Asteraceae
Tilia	*Tilia cordata*	Malvaceae
Trifolium pratense	*Trifolium pratense*	Fabaceae
Trifolium repens-group	*Trifolium repens*	Fabaceae
	Trifolium arvense	Fabaceae

	Trifolium campestre	Fabaceae
	Trifolium dubium	Fabaceae
	Trifolium fragiferum	Fabaceae
Viola arvensis-group	*Viola arvensis*	Violaceae
	Viola tricolor	Violaceae
Solanum	*Solanum dulcamara*	Solanaceae
	Solanum lycopersicum	Solanaceae
Spiraea	*Spiraea x vanhouttei*	Rosaceae
	Spiraea salicifolia	Rosaceae
