# Supplementary material for: Bumblebee flower constancy and pollen diversity over time
Source: Behav Ecol. 2023 Apr 10;34(4):602–12. doi: 10.1093/beheco/arad028 (PMC10332455; doi:10.1093/beheco/arad028)
Supplement: arad028_suppl_Supplementary_Appendix_D [file arad028_suppl_supplementary_appendix_d.pdf]

Appendix D. Qqplot of model residuals and graph with residual frequencies and outliers marked out, based on the pollen load similarity model. The graphs were produced with the DHARMa package (Hartig 2018) in R.

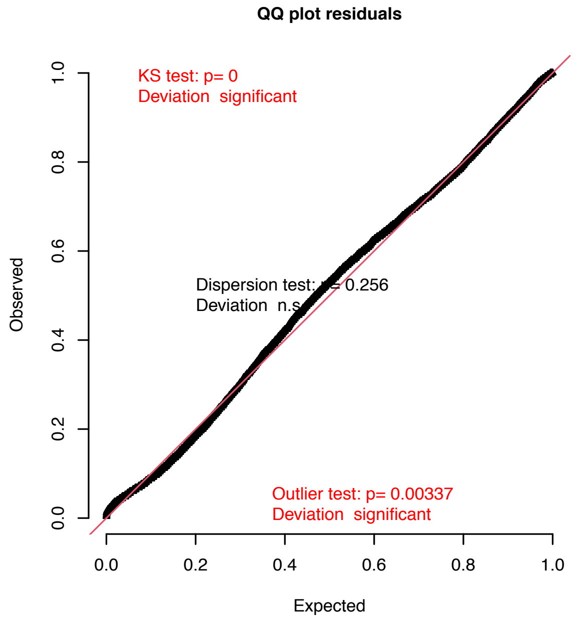

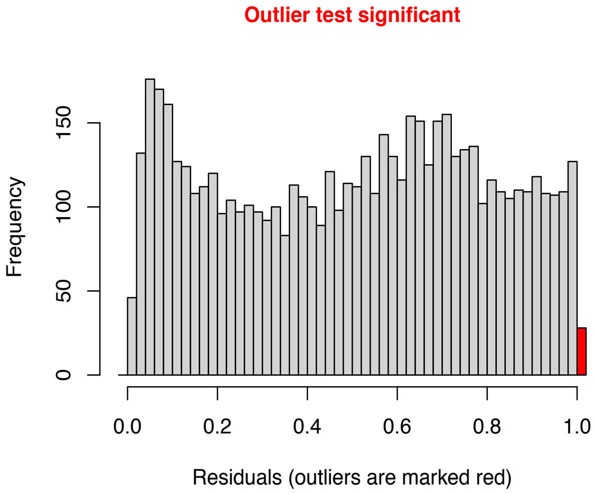

Reference:

Hartig, F. 2018. DHARMa: Residual Diagnostics for Hierarchical (Multi-Level / Mixed) Regression Models. Page R package version 0.2.0.
